# Adaptive Consensus-Based Unscented Information Filter for Tracking Target with Maneuver and Colored Noise

**DOI:** 10.3390/s19143069

**Published:** 2019-07-11

**Authors:** Zhao Li, Yidi Wang, Wei Zheng

**Affiliations:** College of Aerospace Science and Engineering, National University of Defense Technology, Changsha 410073, China

**Keywords:** target tracking, distributed estimation, consensus strategy, information filter, sensor network

## Abstract

Distributed state estimation plays a key role in space situation awareness via a sensor network. This paper proposes two adaptive consensus-based unscented information filters for tracking target with maneuver and colored measurement noise. The proposed filters can fulfill the distributed estimation for non-linear systems with the aid of a consensus strategy, and can reduce the impact of colored measurement noise by employing the state augmentation and measurement differencing methods. In addition, a fading factor that shrinks the predicted information state and information matrix can suppress the impact of dynamical model error induced by target maneuvers. The performances of the proposed algorithms are investigated by considering a target tracking problem using a space-based radar network. This shows that the proposed algorithms outperform the traditional consensus-based distributed state estimation method in aspects of tracking stability and accuracy.

## 1. Introduction

With the development of sensor technology, wireless sensor networks (WSN) are widely used in various fields, including target tracking, industrial automation and cognitive radio communication systems [1,2,3]. Specifically, the performance of space target tracking can be significantly improved by using WSN, such as radar sensor networks and optical sensor networks [4,5]. The state estimation algorithm is essential for target tracking, and can be roughly divided into two categories: the centralized algorithm and the distributed one [6]. In centralized state estimation, the measurements from all sensors are sent to a fusion center, so the computation and communication burden imposed on the fusion center are severe. By contrast, the distributed state estimation algorithm does not require any fusion center, and the information exchange only takes place between the neighboring nodes. So the distributed method has good scalability, low computation burden, and is robust to the failure of the sensor node, which has made it a research hotspot in recent years [7,8].

In the distributed estimation field, the consensus-based algorithm draws much attention because it has global convergence properties and is easy to implement [9,10,11,12,13]. The most commonly used are consensus-based Kalman filter (CKF) and consensus-based information filter (CIF) [14,15]. Compared with CKF, CIF has a higher computational efficiency and is more suitable for distributed target tracking [16,17]. Furthermore, in order to achieve the distributed state estimation in non-linear systems, the consensus-based extend information filter (CEIF) and consensus-based unscented information filter (CUIF) are proposed [18]. Compared with CEIF, CUIF is more accurate because unscented transformation is used to approximate the posterior mean and covariance of random state variables, and it is easier to adopt because the derivation of Jacobian matrices is not required.

The space-based radar tracking must take the colored measurement noise into consideration [19]. The colored measurement noise in radar systems is caused by the scintillation of the target [20], and its impact cannot be ignored [21]. So far, the previous distributed state estimation methods used in space target tracking did not handle this unfavorable factor. For the distributed linear estimation problem, [22] two methods were applied, which include the state augmentation approach and the measurement differencing approach [23,24,25], to cope with the colored measurement noise which was modeled as an autoregressive model.

Dynamical model error also has a significant impact on the tracking performance of space-based radar system. In this case, the dynamical model error is mainly caused by the orbit maneuver of target. The traditional state estimation algorithms might diverge if the dynamical model used by the filter does not reflect the real motion of a target during a maneuver [26]. The interacting multiple model (IMM) is often applied in maneuvering target tracking [27]. In IMM, a model set including several models is used to deal with the varying characteristics of target motion. For distributed target tracking, an adaptive interacting multiple model (AIMM) has been employed in CUIF to improve the tracking accuracy for a maneuvering target [28]. However, the IMM and AIMM methods both are based on the assumption that the target motion evolves according to some predetermined models. So they are not suitable for space target tracking because the orbital maneuvers are usually implemented by impulsive thrust which cannot be modeled. Another effective method to reduce the impact of dynamical model error is to inflate the predicted state covariance through a fading factor [29,30]. This method has been applied in the unscented Kalman filter to track a space maneuvering target [31].

In this paper, we propose two adaptive consensus-based non-linear information filters for tracking target with maneuver and colored measurement noise. The state augmentation and measurement differencing approaches are employed to deal with the colored noise in a discrete-time non-linear system. And the fading factor is used in the framework of distributed information filter to reduce the impact of dynamical model error during the orbital maneuver. The performances of proposed algorithms are compared in the cases where a space target is tracked in a space-based radar network. The simulation results show the proposed filters can effectively track the maneuvering target and achieve higher tracking accuracy than CUIF. 

The remaining part of the paper is organized as follows. Section 2 describes the system model of space target tracking. Section 3 clarifies the fundamentals of the proposed filter. Section 4 presents the proposed consensus-based distributed filters for tracking target with maneuver and colored measurement noise. The tracking performances of proposed methods are demonstrated by simulations in Section 5. Finally, the main conclusions are given in Section 6.

## 2. Space Target Tracking System Model

The space target tracking system model includes the orbital dynamical model and the measurement model, and it is used as a reference for the implementation of the filter in Section 3. The orbital dynamical model takes $J_{2}$ non-spherical perturbation into account because it is the most influential perturbation. The microwave radar can provide range measurements between the observation platform and the target, and its measurement model is given in the following section.

### 2.1. The Orbital Dynamical Model

An Earth-centered inertial coordinate system is selected, and the orbital dynamical model of a space object in a low Earth orbit (LEO) is given by [32]:

(1) $$\left[\begin{array}{c} \dot{r}\\ \dot{v} \end{array}\right]=\left[\begin{array}{c} v\\ a \end{array}\right]+\left[\begin{array}{c} w_{v}\\ w_{a} \end{array}\right],$$

(2) $$a=a_{T}+a_{J_{2}},$$

(3) $$a_{T}=-\mu_{e}\frac{r}{r^{3}},$$

(4) $$a_{J_{2}}=\frac{3}{2}J_{2}{\left(\frac{R_{e}}{r}\right)}^{2}\left(\frac{\mu_{e}}{r^{3}}\right)\left[\begin{array}{c} x\left(5\frac{z}{r^{2}}-1\right)\\ y\left(5\frac{z}{r^{2}}-1\right)\\ z\left(5\frac{z}{r^{2}}-3\right) \end{array}\right],$$

in which, r and v are the position and velocity of the target, $r={\left[x,\;y,\;z\right]}^{T}$ and $r=\sqrt{x^{2}+y^{2}+z^{2}}$; a is the acceleration of the target, $a_{T}$ is the two-body central gravitational acceleration, $a_{J_{2}}$ is the $J_{2}$ non-spherical perturbation acceleration, $J_{2}\approx 0.00108263$; $w={\left[w_{v},w_{a}\right]}^{T}$ is the process noise, $\mu_{e}$ is the Earth gravitational constant, and $R_{e}$ is the Earth radius. 

### 2.2. The Measurement Model

The measurement model of microwave radar is given by:

(5) $$\rho_{k}^{i}=\left\|r_{k}-r_{k}^{i}\right\|=\sqrt{{\left(x_{k}-x_{k}^{i}\right)}^{2}+{\left(y_{k}-y_{k}^{i}\right)}^{2}+{\left(z_{k}-z_{k}^{i}\right)}^{2}}+v_{k}^{i},$$

where $r_{k}$ is the position of the target at *k*th time step, $r_{k}^{i}$ is the position of the *i*th radar, and $v_{k}^{i}$ is the measurement noise of the *i*th radar. The measurement noise $v_{k}^{i}$ is colored noise because it is correlated with itself at different time steps. From the previous study [33], we can know that the first order auto regressive (AR) model can describe the time-varying characteristics of colored noise well. So the time-varying characteristic of $v_{k}^{i}$ is described by the following first-order AR model:

(6) $$v_{k}^{i}=a_{k}^{i}v_{k-1}^{i}+\varepsilon_{k}^{i},$$

in which, $a_{k}^{i}$ is the known correlation parameter; $\varepsilon_{k}$ is zero-mean Gaussian white noise which is uncorrelated with $v_{k-1}^{i}$, and its covariance is $R_{k}^{i}=\sigma_{\rho }^{2}$. This noise model is used to build the methods for handling colored measurement noise in Section 3.2.

## 3. Fundamentals of the Proposed Filter

### 3.1. Brief Review of Consensus-Based State Estimation Algorithm

Let us consider the following non-linear discrete-time dynamical system:

(7) $$x_{k}=f(x_{k-1})+w_{k-1},$$

(8) $$z_{k}^{i}=h^{i}(x_{k})+v_{k}^{i},\;i=1,2,\cdots N,$$

where the dynamical model and measurement model are represented by (7) and (8) respectively; $x_{k}$ represents the system state at time *k*, and $x_{k}={\left[\begin{array}{cc} r_{k} & v_{k} \end{array}\right]}^{T}$; $z_{k}^{i}$ is the measurement by the *i*th radar at time *k*, and $z_{k}^{i}=\rho_{k}^{i}$; N is the number of radars; $w_{k-1}$ is zero-mean Gaussian white noise with covariance $Q_{k-1}$, and the process noise and the measurement noise are uncorrelated. The communication topology of the radar network can be described by an undirected graph G=(C,E), where C={1,2,⋯,N} is the vertex set and E={(i,j)|i,j∈C} is the edge set. The neighbor node of the *i*th node is defined as $N_{i}=\left\{j\in C|\left(i,j\right)\in E\right\}$, which has $N_{i}$ nodes.

According to [18,34], the process of the CUIF is summarized in Algorithm 1. In which, *L* is the number of consensus iterations; θ is the consensus rate and $0<\theta <1/\Delta_{\max}$, where $\Delta_{\max}$ is the maximum degree of the graph. 

Assuming that the predicted state $x_{k|k-1}^{i}$ and the associated covariance matrix $P_{k|k-1}^{i}$ at *k*th time step for *i*th local filter are known, for the space target tracking problem considered in this paper, the initial state and the associated covariance matrix of the target can be estimated from few measurements by an initial orbit determination method proposed in [35]. This supposes that the target does not operate orbital maneuvers before the tracking. So $x_{k|k-1}^{i}$ and $P_{k|k-1}^{i}$ are obtained by orbital prediction based on the orbital dynamical model. Then the predicted information state and the information matrix are calculated by:

(9) $$y_{k|k-1}^{i}={\left(P_{k|k-1}^{i}\right)}^{-1}x_{k|k-1}^{i},$$

(10) $$Y_{k|k-1}^{i}={\left(P_{k|k-1}^{i}\right)}^{-1}.$$

**Algorithm 1** Consensus-based unscented information filter (CUIF)**Step 1.** Compute consensus proposals of local filter:
(11)$$v_{k,0}^{i}=\frac{1}{N}y_{k|k-1}^{i}+\phi_{k}^{i},$$

(12)$$V_{k,0}^{i}=\frac{1}{N}Y_{k|k-1}^{i}+\Phi_{k}^{i},$$
**Step 2.** Perform consensus on $v_{k,0}^{i}$ and $V_{k,0}^{i}$  **for**
*l* = 1 to *L*    I. Send $v_{k,l-1}^{i}$ and $V_{k,l-1}^{i}$ to all neighbors;    II. Receive $v_{k,l-1}^{j}$ and $V_{k,l-1}^{j}$ from all neighbors;    III. Update consensus terms:
(13)$$v_{k,l}^{i}=v_{k,l-1}^{i}+\theta \sum_{j\in N_{i}}\left(v_{k,l-1}^{j}-v_{k,l-1}^{i}\right),$$

(14)$$V_{k,l}^{i}=V_{k,l-1}^{i}+\theta \sum_{j\in N_{i}}\left(V_{k,l-1}^{j}-V_{k,l-1}^{i}\right),$$
  **end for****Step 3.** Compute the posterior at *k*th time step:
(15)$$y_{k}^{i}=Nv_{k,L}^{i},\;Y_{k}^{i}=NV_{k,L}^{i},$$

(16)$${\hat{x}}_{k}^{i}={\left(Y_{k}^{i}\right)}^{-1}y_{k}^{i},\;P_{k}^{i}={\left(Y_{k}^{i}\right)}^{-1},$$
**Step 4.** Prediction for the next time step:  The predicted system state and its covariance matrix are obtained by:
(17)$$\chi_{\tau ,k+1|k}^{i}=f\left(\chi_{\tau ,k}^{i}\right).$$

(18)$$x_{k+1|k}^{i}=\sum_{\tau =0}^{2n}\omega_{\tau }^{m}\chi_{\tau ,k+1|k}^{i},$$

(19)$$P_{k+1|k}^{i}=\sum_{\tau =0}^{2n}\omega_{\tau }^{c}\left[\chi_{\tau ,k+1|k}^{i}-x_{k+1|k}^{i}\right]{\left[\chi_{\tau ,k+1|k}^{i}-x_{k+1|k}^{i}\right]}^{T}+Q_{k}.$$


In (11) and (12), $\phi_{k}^{i}$ and $\Phi_{k}^{i}$ are the contributions of the information state and matrix, respectively. They are obtained by:

(20) $$f_{k}^{i}=\left(Y_{k|k-1}^{i}P_{xz,k}^{i}\right){\left(R_{k}^{i}\right)}^{-1}\left(z_{k}^{i}-z_{k|k-1}^{i}+{\left(P_{xz,k}^{i}\right)}^{T}y_{k|k-1}^{i}\right),$$

(21) $$\Phi_{k}^{i}=\left(Y_{k|k-1}^{i}P_{xz,k}^{i}\right){\left(R_{k}^{i}\right)}^{-1}{\left(Y_{k|k-1}^{i}P_{xz,k}^{i}\right)}^{T},$$

and

(22) $$z_{\tau ,k|k-1}^{i}=h\left(\chi_{\tau ,k|k-1}^{i}\right),$$

(23) $$z_{k|k-1}^{i}=\sum_{\tau =0}^{2n}\omega_{\tau }^{m}z_{\tau ,k|k-1}^{i},$$

(24) $$P_{xz,k}^{i}=\sum_{\tau =0}^{2n}\omega_{\tau }^{c}\left[\chi_{\tau ,k|k-1}^{i}-x_{k|k-1}^{i}\right]{\left[z_{\tau ,k|k-1}^{i}-z_{k|k-1}^{i}\right]}^{T},$$

In (16), $\chi_{\tau ,k}^{i}$ is the set of sigma point, and

(25) $$\{\begin{array}{l} \chi_{0,k}^{i}={\hat{x}}_{k}^{i},\\ \chi_{\tau ,k}^{i}={\hat{x}}_{k}^{i}+\sqrt{n+\lambda }\cdot {\left(\sqrt{P_{k}^{i}}\right)}_{\tau },\;\tau =1,2,\cdots ,n\\ \chi_{\tau +n,k}^{i}={\hat{x}}_{k}^{i}-\sqrt{n+\lambda }\cdot {\left(\sqrt{P_{k}^{i}}\right)}_{\tau },\;\tau =n+1,n+2,\cdots ,2n, \end{array}$$

where $\lambda =\alpha^{2}(n+K)-n$, α is used to control the distribution of sigma points and 0<α<1, and K is equal to 3-n; ${\left(\sqrt{P_{k}^{i}}\right)}_{\tau }$ is the τth row for the Cholesky factor of $P_{k}^{i}$.

In (18) and (19), $\omega_{\tau }^{m}$ is the weighted value corresponding to each sigma point, and $\omega_{\tau }^{c}$ is the weighted values corresponding to variance matrix. They can be obtained as follows:

(26) $$\{\begin{array}{l} \omega_{0}^{m}=\frac{\lambda }{n+\lambda },\\ \omega_{0}^{c}=\frac{\lambda }{n+\lambda }+\left(1-\alpha^{2}+\beta \right),\\ \omega_{\tau }^{m}=\omega_{\tau }^{c}=\frac{1}{2\left(n+\lambda \right)},\;\tau =1,\cdots ,2n, \end{array}$$

where β is the parameter related with the prior distribution of state and is set as 2 generally for Gaussian distribution. 

### 3.2. Method for Handling Colored Measurement Noise

#### 3.2.1. State Augmentation

The state augmentation is a simple way to deal with the colored measurement noise. This method is applied in distributed state estimation for a linear discrete-time system in [22]. In this section, we will extend it to a non-linear system, and the dynamical model (7) and the measurement model (8) are rewritten as follows.

The dynamical model with the augmented state for each radar node is given by:

(27) $${x^{\prime }}_{k}^{i}=f^{\prime }\left({x^{\prime }}_{k-1}^{i}\right)+{w^{\prime }}_{k}^{i},$$

where

(28) $${x^{\prime }}_{k}^{i}=\left[\begin{array}{c} x_{k}^{i}\\ v_{k}^{i} \end{array}\right],$$

(29) $$f^{\prime }\left({x^{\prime }}_{k-1}^{i}\right)=\left[\begin{array}{c} f\left(x_{k-1}^{i}\right)\\ 0 \end{array}\right]+a_{k}^{i}\left[\begin{array}{c} 0\\ v_{k-1}^{i} \end{array}\right],$$

(30) $${w^{\prime }}_{k}^{i}=\left[\begin{array}{c} w_{k-1}\\ \varepsilon_{k}^{i} \end{array}\right],$$

and

(31) $${Q^{\prime }}_{k}=E\left[{w^{\prime }}_{k}^{i}{\left({w^{\prime }}_{k}^{i}\right)}^{T}\right]=\left[\begin{array}{cc} Q_{k} & 0\\ 0 & R_{k}^{i} \end{array}\right],$$

Then, the measurement model for each node becomes:

(32) $$z_{k}^{i}={h^{\prime }}^{i}\left({x^{\prime }}_{k}^{i}\right)+{v^{\prime }}_{k}^{i}=\left[\begin{array}{cc} 1 & 1 \end{array}\right]\cdot \left[\begin{array}{c} h^{i}\left(x_{k}^{i}\right)\\ v_{k}^{i} \end{array}\right],$$

where ${v^{\prime }}_{k}^{i}=0$, so

(33) $${R^{\prime }}_{k}^{i}=E\left[{v^{\prime }}_{k}^{i}{\left({v^{\prime }}_{k}^{i}\right)}^{T}\right]=0,$$

from the above equations, we know the measurement of the augmented system does not contain noise, and hence, it can cause the ill-conditioned problem in the calculation of (20) and (21). So the value of ${R^{\prime }}_{k}^{i}$ is set as $0.3*R_{k}^{i}$ to ensure that the filter can work.

#### 3.2.2. Measurement Differencing

Moreover, the measurement differencing can also convert the colored measurement noise into white noise. The contribution of the colored portion of the measurement noise is subtracted by constructing new measurements. Compared with the state augmentation method, the measurement differencing method has a lower dimensionality, and the risk of ill-conditioned calculations can be avoided. The specific implementation process of the measurement differencing method is as follows.

For each radar node, an auxiliary measurement is defined by:

(34) $${\tilde{z}}_{k}^{i}=z_{k}^{i}-a_{k}^{i}z_{k-1}^{i},$$

substituting $z_{k}^{i}$ and $z_{k-1}^{i}$ into (34), we can obtain:

(35) $$\begin{array}{l} {\tilde{z}}_{k}^{i}=h^{i}\left(x_{k}^{i}\right)+v_{k}^{i}-a_{k}^{i}\left(h^{i}\left(x_{k-1}^{i}\right)+v_{k-1}^{i}\right)\\ \;=h^{i}\left(f\left(x_{k-1}^{i}\right)+w_{k-1}\right)+v_{k}^{i}-a_{k}^{i}\left(h^{i}\left(x_{k-1}^{i}\right)+v_{k-1}^{i}\right)\\ \;=h^{i}\left(f\left(x_{k-1}^{i}\right)\right)-a_{k}^{i}h^{i}\left(x_{k-1}^{i}\right)+h^{i}\left(w_{k-1}\right)+v_{k}^{i}-a_{k}^{i}v_{k-1}^{i}\\ \;=h^{i}\left(f\left(x_{k-1}^{i}\right)\right)-a_{k}^{i}h^{i}\left(x_{k-1}^{i}\right)+h^{i}\left(w_{k-1}\right)+\varepsilon_{k}^{i} \end{array}$$

Then we have an equivalent system for target tracking, and its dynamical model is the same as (7) and its measurement model is given by:

(36) $${\tilde{z}}_{k}^{i}={\tilde{h}}^{i}\left(x_{k-1}^{i}\right)+{\tilde{v}}_{k}^{i},$$

where

(37) $${\tilde{h}}^{i}\left(x_{k-1}^{i}\right)=h^{i}\left(f\left(x_{k-1}^{i}\right)\right)-a_{k}^{i}h^{i}\left(x_{k-1}^{i}\right),$$

(38) $${\tilde{v}}_{k}^{i}=h^{i}\left(w_{k-1}\right)+\varepsilon_{k}^{i},$$

in this equivalent system, the time-correlated portion of the measurement noise $v_{k}^{i}$ does not appear in the new measurement ${z^{\prime }}_{k}^{i}$. The measurement noise ${v^{\prime }}_{k}^{i}$ in ${z^{\prime }}_{k}^{i}$ is a zero-mean Gaussian white noise. Therefore, the covariance of the new measurement noise ${v^{\prime }}_{k}^{i}$, and the cross-covariance between the process noise and the new measurement noise are obtained by:

(39) $$\begin{array}{l} E\left[{\tilde{v}}_{k}^{i}{\left({\tilde{v}}_{k}^{i}\right)}^{T}\right]=E\left[\left(h^{i}\left(w_{k-1}\right)+\varepsilon_{k}^{i}\right){\left(h^{i}\left(w_{k-1}\right)+\varepsilon_{k}^{i}\right)}^{T}\right]\\ \;=H_{k}^{i}Q_{k}{\left(H_{k}^{i}\right)}^{T}+R_{k}^{i} \end{array}$$

(40) $$\begin{array}{l} E\left[w_{k}{\left({\tilde{v}}_{k}^{i}\right)}^{T}\right]=E\left[w_{k}{\left(h^{i}\left(w_{k-1}\right)+\varepsilon_{k}^{i}\right)}^{T}\right]\\ \;=Q_{k}{\left(H_{k}^{i}\right)}^{T} \end{array}$$

where $H_{k}^{i}$ is the Jacobian matric of the measurement function $h^{i}$, and

(41) $$H_{k}^{i}={\frac{\partial h^{i}\left(x\right)}{\partial x}|}_{x=x_{k/k-1}^{i}}$$

### 3.3. Method for Handling Dynamical Model Error

According to the method used in [31], the $P_{k|k-1}^{i}$ is inflated by a fading factor in order to reduce the contribution of $x_{k|k-1}^{i}$ to ${\hat{x}}_{i,k}$ during the maneuver. In this way, the impact of the dynamical model error caused by maneuvers on the tracking accuracy is reduced. This method for handling dynamical model error has been employed in the framework of the centralized Kalman filter, and we will extend it to the distributed information filter. 

The fading factor is obtained by:

(42) $$\alpha_{k}^{i}=\left\{ \begin{array}{l} \alpha_{k,0}^{i}\;\alpha_{k,0}^{i}>1\\ \;1\;\alpha_{k,0}^{i}\leq 1, \end{array}\right.$$

(43) $$\alpha_{k,0}^{i}=tr\left[C_{k}^{i}-R_{k}^{i}\right]/tr\left[P_{z_{i}z_{i},k}-R_{k}^{i}\right],$$

and

(44) $$C_{k}^{i}=\left\{ \begin{array}{l} \;\gamma_{k}^{i}\gamma_{k}^{i\;T}\;k=1\\ \frac{\lambda C_{k-1}^{i}+\gamma_{k}^{i}\gamma_{k}^{i\;T}}{1+\lambda }\;k>1, \end{array}\right.$$

where λ is the forgetting factor commonly determined to be 0.95.

In the Kalman filer, the fading factor is added by the following equations:

(45) $${\overset{⌣}{P}}_{k|k-1}^{i}=\alpha_{k}^{i}P_{k|k-1}^{i},$$

(46) $${\overset{⌣}{P}}_{z_{i}z_{i},k}=\alpha_{k}^{i}P_{z_{i}z_{i},k},$$

(47) $${\overset{⌣}{P}}_{xz,k}^{i}=\alpha_{k}^{i}P_{xz,k}^{i},$$

substituting (45) into (9) and (10), we know that the predicted information state and the information matrix after adding the fading factor is calculated by:

(48) $${\overset{⌣}{y}}_{k|k-1}^{i}={\left({\overset{⌣}{P}}_{k|k-1}^{i}\right)}^{-1}x_{k|k-1}^{i}=\frac{1}{\alpha_{k}^{i}}y_{k|k-1}^{i},$$

(49) $${\overset{⌣}{Y}}_{k|k-1}^{i}={\left({\overset{⌣}{P}}_{k|k-1}^{i}\right)}^{-1}=\frac{1}{\alpha_{k}^{i}}Y_{k|k-1}^{i},$$

and the contributions of the information state is given by:

(50) $$\begin{array}{l} {\overset{⌣}{\phi }}_{k}^{i}=\left({\overset{⌣}{Y}}_{k|k-1}^{i}{\overset{⌣}{P}}_{xz,k}^{i}\right){\left(R_{k}^{i}\right)}^{-1}\left(z_{k}^{i}-z_{k|k-1}^{i}+{\left({\overset{⌣}{P}}_{xz,k}^{i}\right)}^{T}{\overset{⌣}{y}}_{k|k-1}^{i}\right)\\ \;=\left[{\left(\alpha_{k}^{i}P_{k|k-1}^{i}\right)}^{-1}\alpha_{k}^{i}P_{xz,k}^{i}\right]{\left(R_{k}^{i}\right)}^{-1}\left[z_{k}^{i}-z_{k|k-1}^{i}+{\left(\alpha_{k}^{i}P_{xz,k}^{i}\right)}^{T}\left({\left(\alpha_{k}^{i}P_{k|k-1}^{i}\right)}^{-1}x_{k|k-1}^{i}\right)\right]\\ \;=\left[{\left(P_{k|k-1}^{i}\right)}^{-1}P_{xz,k}^{i}\right]{\left(R_{k}^{i}\right)}^{-1}\left[z_{k}^{i}-z_{k|k-1}^{i}+{\left(P_{xz,k}^{i}\right)}^{T}\left({\left(P_{k|k-1}^{i}\right)}^{-1}x_{k|k-1}^{i}\right)\right]\\ \;=\phi_{k}^{i} \end{array}$$

in the same way, there is

(51) $${\overset{⌣}{\Phi }}_{k}^{i}=\Phi_{k}^{i}.$$

So the consensus proposals computing step of the CUIF is rewritten as:

(52) $$v_{k,0}^{i}=\frac{1}{N}{\overset{⌣}{y}}_{k|k-1}^{i}+{\overset{⌣}{\phi }}_{k}^{i}=\frac{1}{N\cdot \alpha_{k}^{i}}y_{k|k-1}^{i}+\phi_{k}^{i},$$

(53) $$V_{k,0}^{i}=\frac{1}{N}{\overset{⌣}{Y}}_{k|k-1}^{i}+{\overset{⌣}{\Phi }}_{k}^{i}=\frac{1}{N\cdot \alpha_{k}^{i}}Y_{k|k-1}^{i}+\Phi_{k}^{i},$$

from the above equations, we can know the predicted information state and matrix are shrunken by the fading factor. In other words, the information provided by the dynamical model is discarded during the maneuver, so the impact of the dynamical model error caused by the maneuver is suppressed.

## 4. Adaptive Consensus-Based Unscented Information Filter

In the framework of CUIF, by using the modified system model (27) and measurement model (32) in the local filter of each radar node, and adding the fading factor, we can obtain the adaptive consensus-based information filter based on state augmentation, which is summarized in Algorithm 2. Assuming that the predicted augmented state ${x^{\prime }}_{k|k-1}^{i}$ and the associated covariance matrix ${P^{\prime }}_{k|k-1}^{i}$ at *k*th time step for *i*th local filter are known. Then, the predicted information state ${y^{\prime }}_{k|k-1}^{i}$ and the information matrix ${Y^{\prime }}_{k|k-1}^{i}$ can be obtained according to (9) and (10). It is worth noting that the consensus is carried out only on the original state, and each radar node runs a local filter with augmented state including its own colored measurement noise $v_{k}^{i}$. This is similar to the strategy used in [22]. Besides, the filter based on measurement differencing is presented in Algorithm 3. By comparing the process of the two algorithms, it can be see that the algorithm based on measurement differencing is easier to implement. In the next section, the performances of these two distributed state estimation algorithms under different conditions are compared.

**Algorithm 2** Adaptive CUIF based on state augmentation (ACUIF-SA)**Step 1.** Compute consensus proposals of the original state for local filter:  The fading factor $\alpha_{k}^{i}$ is calculated according to (42)–(44), then $v_{k,0}^{i}$ and $V_{k,0}^{i}$ are calculated by
(54)$$v_{k,0}^{i}=\frac{1}{N\cdot \alpha_{k}^{i}}y_{k|k-1}^{i}+\phi_{k}^{i},$$

(55)$$V_{k,0}^{i}=\frac{1}{N\cdot \alpha_{k}^{i}}Y_{k|k-1}^{i}+\Phi_{k}^{i},$$
where ${\phi^{\prime }}_{k}^{i}$ and ${\Phi^{\prime }}_{k}^{i}$ are obtained by:
(56)$$\phi_{k}^{i}=\left(Y_{k|k-1}^{i}P_{xz,k}^{i}\right){\left({R^{\prime }}_{k}^{i}\right)}^{-1}\left(z_{k}^{i}-{z^{\prime }}_{k|k-1}^{i}+{\left(P_{xz,k}^{i}\right)}^{T}y_{k|k-1}^{i}\right),$$

(57)$$\Phi_{k}^{i}=\left(Y_{k|k-1}^{i}P_{xz,k}^{i}\right){\left({R^{\prime }}_{k}^{i}\right)}^{-1}{\left(Y_{k|k-1}^{i}P_{xz,k}^{i}\right)}^{T},$$
and
(58)$$P_{xz,k}^{i}=\sum_{\tau =0}^{2n}\omega_{\tau }^{c}\left[\chi_{\tau ,k|k-1}^{i}-x_{k|k-1}^{i}\right]{\left[{z^{\prime }}_{\tau ,k|k-1}^{i}-{z^{\prime }}_{k|k-1}^{i}\right]}^{T},$$

(59)$${z^{\prime }}_{\tau ,k|k-1}^{i}={h^{\prime }}^{i}\left({\chi^{\prime }}_{\tau ,k|k-1}^{i}\right),$$
in which, ${h^{\prime }}^{i}$ is given by (32).**Step 2.** Perform consensus on $v_{k,0}^{i}$ and $V_{k,0}^{i}$ via (13) and (14).**Step 3.** Compute the posterior at *k*th time step:  (1) Compute the posterior of the original state at *k*th time step according to (15) and (16), ${\hat{x}}_{k}^{i}$ and $P_{k}^{i}$ can be obtained;   (2) Compute the posterior of the augmented state and the associated covariance matrix by:
(60)$${{\hat{x}}^{\prime }}_{k,0}^{i}={\left({Y^{\prime }}_{k}^{i}\right)}^{-1}{y^{\prime }}_{k}^{i},\;{P^{\prime }}_{k,0}^{i}={\left({Y^{\prime }}_{k}^{i}\right)}^{-1},$$
and
(61)$${y^{\prime }}_{k}^{i}={y^{\prime }}_{k|k-1}^{i}+\left({Y^{\prime }}_{k|k-1}^{i}{P^{\prime }}_{xz,k}^{i}\right){\left({R^{\prime }}_{k}^{i}\right)}^{-1}\left(z_{k}^{i}-{z^{\prime }}_{k|k-1}^{i}+{\left({P^{\prime }}_{xz,k}^{i}\right)}^{T}{y^{\prime }}_{k|k-1}^{i}\right),$$

(62)$${Y^{\prime }}_{k}^{i}={Y^{\prime }}_{k|k-1}^{i}+\left({Y^{\prime }}_{k|k-1}^{i}{P^{\prime }}_{xz,k}^{i}\right){\left({R^{\prime }}_{k}^{i}\right)}^{-1}{\left({Y^{\prime }}_{k|k-1}^{i}{P^{\prime }}_{xz,k}^{i}\right)}^{T},$$

(63)$${P^{\prime }}_{xz,k}^{i}=\sum_{\tau =0}^{2n}\omega_{\tau }^{c}\left[{\chi^{\prime }}_{\tau ,k|k-1}^{i}-{x^{\prime }}_{k|k-1}^{i}\right]{\left[{z^{\prime }}_{\tau ,k|k-1}^{i}-{z^{\prime }}_{k|k-1}^{i}\right]}^{T},$$
  (3) Reset the local filter:
(64)$${{\hat{x}}^{\prime }}_{k}^{i}=={\left[\begin{array}{cc} {\hat{x}}_{k}^{i} & {\hat{v}}_{k}^{i} \end{array}\right]}^{T},\;{P^{\prime }}_{k}^{i}=\left[\begin{array}{cc} P_{k}^{i} & 0\\ 0 & P_{v_{k}^{i}} \end{array}\right],$$
where ${\hat{v}}_{k}^{i}={{\hat{x}}^{\prime }}_{k,0}^{i}\left(n+1\right)$, $P_{v_{k}^{i}}={P^{\prime }}_{k,0}^{i}\left(n+1,\;n+1\right)$ and *n* is the dimension of the original state.**Step 4.** Prediction for the next time step:   The predicted augmented state and its covariance matrix can be calculate in a similar way shown in (18) and (19).

**Algorithm 3** Adaptive CUIF based on measurement differencing (ACUIF-MD)**Step 1.** Compute consensus proposals of local filter:  The fading factor $\alpha_{k}^{i}$ is calculated according to (42)–(44), then $v_{k,0}^{i}$ and $V_{k,0}^{i}$ are calculated by:
(65)$$v_{k,0}^{i}=\frac{1}{N\cdot \alpha_{k}^{i}}y_{k|k-1}^{i}+\phi_{k}^{i},$$

(66)$$V_{k,0}^{i}=\frac{1}{N\cdot \alpha_{k}^{i}}Y_{k|k-1}^{i}+\Phi_{k}^{i},$$
where $\phi_{k}^{i}$ and $\Phi_{k}^{i}$ are obtained by:
(67)$$\phi_{k}^{i}=\left(Y_{k|k-1}^{i}{\tilde{P}}_{xz_{i},k}\right){\left(H_{k}^{i}Q_{k}{\left(H_{k}^{i}\right)}^{T}+R_{k}^{i}\right)}^{-1}\left({\tilde{z}}_{k}^{i}-{\tilde{z}}_{k|k-1}^{i}+{\tilde{P}}_{xz_{i},k}^{T}y_{k|k-1}^{i}\right),$$

(68)$$\Phi_{k}^{i}=\left(Y_{k|k-1}^{i}{\tilde{P}}_{xz_{i},k}\right){\left(H_{k}^{i}Q_{k}{\left(H_{k}^{i}\right)}^{T}+R_{k}^{i}\right)}^{-1}{\left(Y_{k|k-1}^{i}{\tilde{P}}_{xz_{i},k}\right)}^{T},$$
and
(69)$${\tilde{z}}_{\;k|k-1}^{i}=\sum_{\tau =0}^{2n}\omega_{\tau }^{m}{\tilde{z}}_{\tau ,k|k-1}^{i},$$

(70)$${\tilde{z}}_{\tau ,k|k-1}^{i}={\tilde{h}}^{i}\left(\chi_{\tau ,k|k-1}^{i}\right),$$

(71)$${\tilde{P}}_{xz,k}^{i}=\sum_{\tau =0}^{2n}\omega_{\tau }^{c}\left[\chi_{\tau ,k|k-1}^{i}-x_{k|k-1}^{i}\right]{\left[{\tilde{z}}_{\tau ,k|k-1}^{i}-{\tilde{z}}_{k|k-1}^{i}\right]}^{T}+Q_{k}{\left(H_{k}^{i}\right)}^{T},$$
in which, ${\tilde{h}}^{i}$ and $H_{k}^{i}$ are given by (37) and (41).**Step 2.** Perform consensus on $v_{k,0}^{i}$ and $V_{k,0}^{i}$ via (13) and (14).**Step 3.** Compute the posterior at *k*th time step according to (15) and (16).**Step 4.** Prediction for the next time step:   Compute the predicted system state and covariance matrix by (18) and (19).

## 5. Simulation Results Analysis

**Figure 1:**
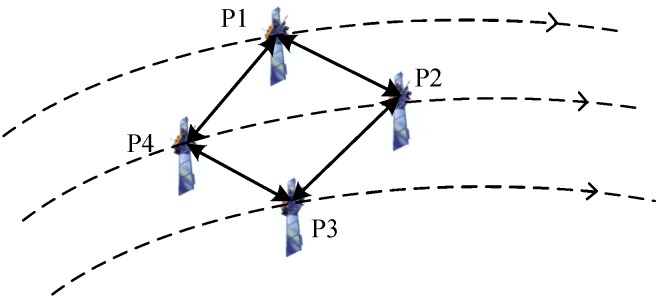
The communication network among the observation platforms.

In this simulation scenario, a LEO target is tracked in a radar network composed of four observation platforms. The initial condition of parameters that would be used in the simulation are listed in Table 1, the initial state covariance matrix and the process noise matrix are defined by (72) and (73). The initial positions and velocities of the target and observation platforms are provided in Table 2. As shown in Figure 1, the communication network topology is denoted by the adjacency matrix (74).

(72) $$P_{0}=\left[\begin{array}{cc} \sigma_{r,0}^{2}E_{3\times 3} & 0\\ 0 & \sigma_{v,0}^{2}E_{3\times 3} \end{array}\right],$$

(73) $$Q_{k}=\left[\begin{array}{cc} \sigma_{v,Q}^{2}E_{3\times 3} & 0\\ 0 & \sigma_{a,Q}^{2}E_{3\times 3} \end{array}\right],$$

(74) $$A=\left[\begin{array}{cccc} 0 & 1 & 0 & 1\\ 1 & 0 & 1 & 0\\ 0 & 1 & 0 & 0\\ 1 & 0 & 1 & 0 \end{array}\right].$$

This section mainly analyses the following two cases: (1) when the measurement noise is colored; (2) when the target operates once orbital maneuver and the measurement noise is colored. For performance comparison, the root mean-squared errors (RMSE) of a position is used as the performance metric. The position RMSE at time *k* is defined by:

(75) $$RMSE_{p}\left(k\right)=\sqrt{\frac{1}{N_{r}}\sum_{n=1}^{N_{r}}\left({\left(x_{k}^{n}-{\hat{x}}_{k}^{n}\right)}^{2}+{\left(y_{k}^{n}-{\hat{y}}_{k}^{n}\right)}^{2}+{\left(z_{k}^{n}-{\hat{z}}_{k}^{n}\right)}^{2}\right)},$$

where $N_{r}$ denotes the number of Monte Carlo runs, and is set as 100 in the following simulations; $\left(x_{k}^{n},y_{k}^{n},z_{k}^{n}\right)$ and $\left({\hat{x}}_{k}^{n},{\hat{y}}_{k}^{n},{\hat{z}}_{k}^{n}\right)$ are the true and estimated positions of the *n*th Monte Carlo run at time *k*. The position RMSE at time *k* is the average of 100 Monte Carlo simulations at the corresponding time.

**Figure 2:**
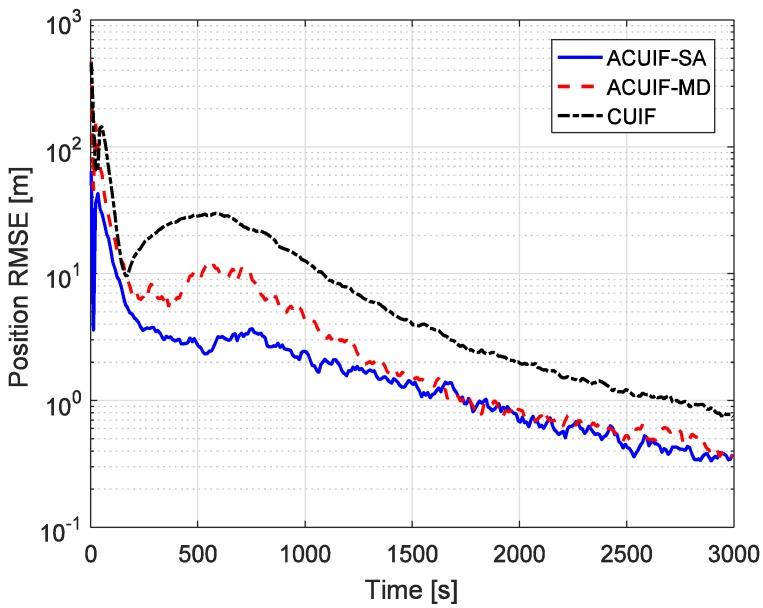
The position root mean-squared error (RMSE) for different methods over time in case 1.

**Figure 3:**
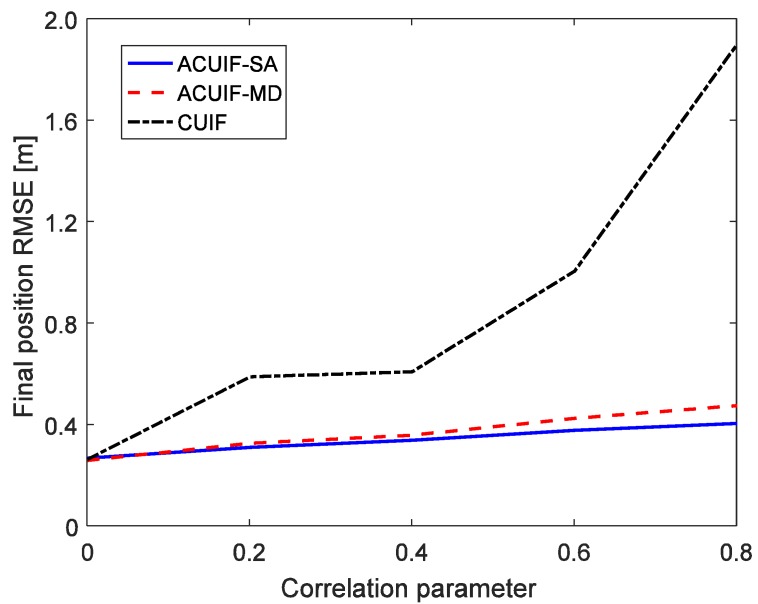
The final position RMSE for the three methods under different colored noises.

In case 1, assuming that the correlation parameter $a_{k}^{i}$ is constant and it is set as 0.5. Figure 2 shows the tracking performance of the ACUIF-SA, ACUIF-MD and CUIF methods in case 1. The abscissa is time, and the ordinate is the RMSE at a certain time instant. It can be seen that the ACUIF-MD and the ACUIF-SA both can achieve higher tracking accuracies than CUIF because the impact of colored noise is surpassed. Besides, the error curves of the ACUIF-SA converges faster than that of the ACUIF-SA, and the final accuracy of the ACUIF-SA is close to that of the ACUIF-SA. Figure 3 shows the final position RMSE of the three filters under different colored noises. The abscissa is correlation parameter of measurement noise, and the ordinate is the RMSE at the last time instant. When the correlation parameter is set as zero, the measurement noise becomes white. The final errors of the three filters are almost the same under this condition. As $a_{k}^{i}$ increases, the influence of the colored measurement noise is more remarkable. It can see that the proposed filters that take the colored the measurement noise into account provide increasingly better performance compared to the traditional CUIF method. And the accuracy of ACUIF-SA is slightly higher than that of ACUIF-MD.

**Figure 4:**
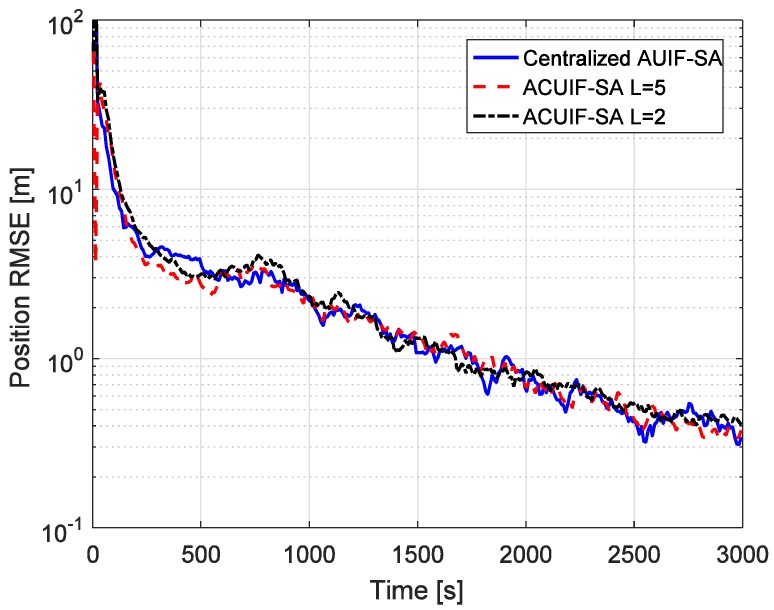
The position RMSE for different methods over time in case 1.

**Figure 5:**
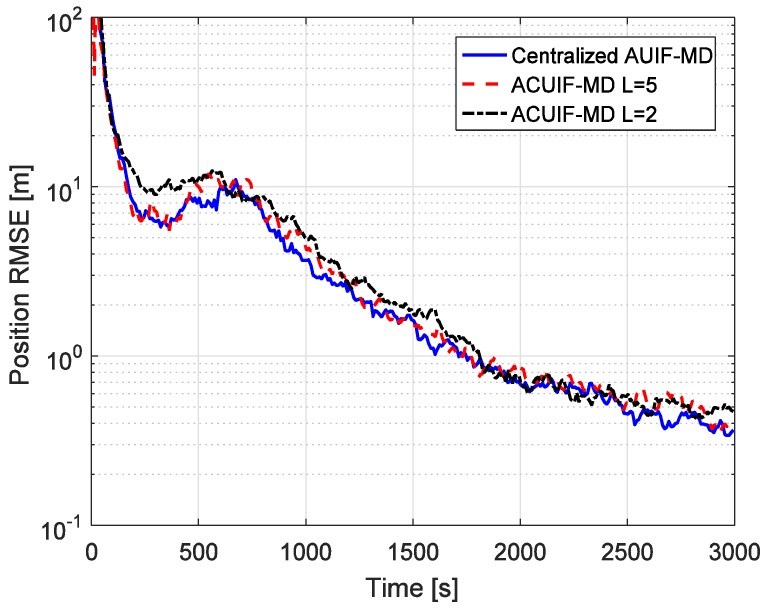
The position RMSE for different methods over time in case 1.

The advantages of the distributed state estimation method make sense only when it can achieve the similar tracking performance as the centralized one. So the tracking performances of the centralized and distributed filters based on state augmentation approach are compared in Figure 4. Centralized AUIF-SA denotes the centralized adaptive unscented information filter based on state augmentation, and the augmented state in this method includes four colored measurement noise terms for different radar nodes. It can be seen from the figure that the accuracy of ACUIF-SA is close to that of centralized AUIF-SA. In addition, the performance degradation of ACUIF-SA is not remarkable when the number of consensus iterations decreases from 5 to 2. This indicates that the proposed distributed filter can work normally when a limited number of consensus iterations is performed. Also, a similar conclusion about the centralized and distributed filters based on the measurement differencing approach can be drawn from Figure 5. 

In case 2, the target operates an orbital maneuver at 1500 s. The corresponding thrust vector is along the direction of the target velocity. And $a_{k}^{i}$ is set as 0.5.

**Figure 6:**
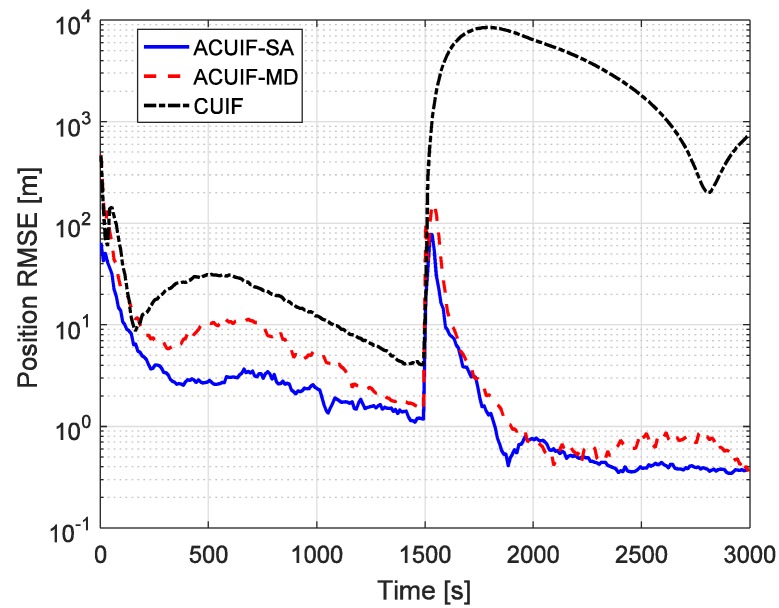
The position RMSE for different methods over time in case 2.

Figure 6 presents the comparison of the tracking performances of the ACUIF-SA, ACUIF-MD and CUIF methods in case 2. The tracking error curves of these three filters display large jumps when the target operates an orbital maneuver. This phenomenon is caused by the dynamical model error between the dynamical model used by the filter and the real one. The CUIF becomes divergent because of the impact of dynamical model error. By contrast, the proposed filters can converge again rapidly. This indicates that the distributed information filter can achieve stable tracking for maneuvering targets by applying the fading factor. Besides, the differences between the final tracking errors of the proposed two filters are not significant, and the convergence rate of the ACUIF-SA is preferable. This is consistent with the conclusion in case 1.

**Figure 7:**
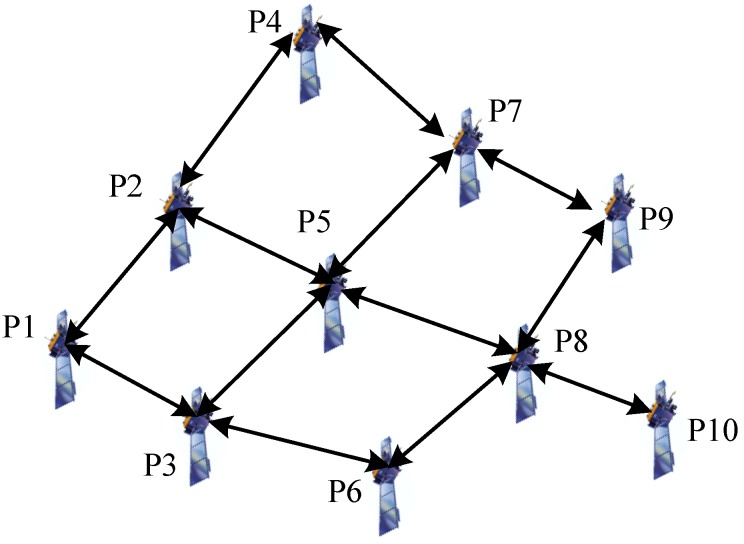
The topology of the sensor network with 10 observation platforms.

**Figure 8:**
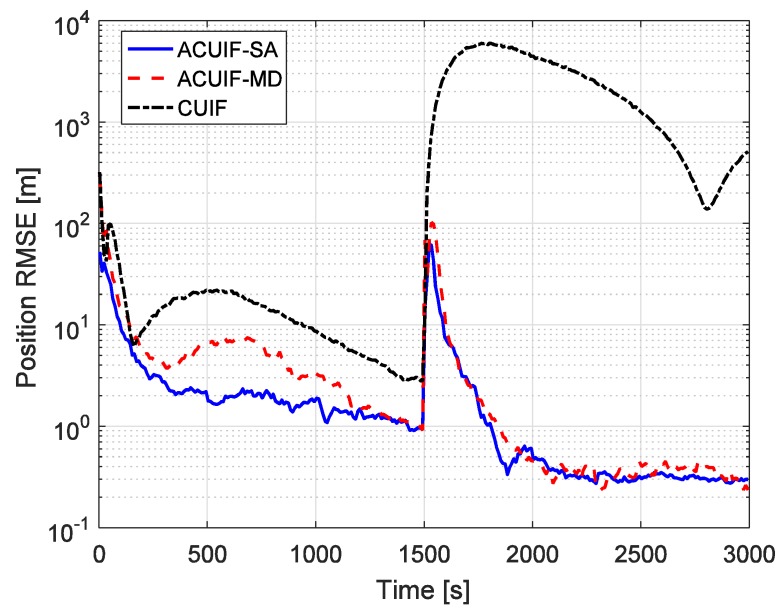
The position RMSE for different methods over time with 10 observation platforms.

Furthermore, in order to show the performance of the proposed methods in a complex network, we have performed a simulation with 10 observation platforms (see Figure 7) and the results are shown in Figure 8. As we can see, the proposed methods can still perform better than the traditional methods in a complex network. Compared with Figure 6, we find that the tracking accuracy can be slightly improved by using more observation platforms.

## 6. Conclusions

In this paper, two adaptive consensus-based unscented information filters are proposed for tracking a target with maneuver and colored measurement noise in a space-based radar network. The state augmentation and measurement differencing approaches are adopted to convert the colored measurement noise into white noise. The fading factor is employed in the framework of a distributed information filter to suppress the impact of the dynamical model error during the orbital maneuver. According to the analysis of the simulation results, the main conclusions are summarized as follows: compared with the traditional CUIF, the ACUIF-SA and ACUIF-MD can track a maneuvering target effectively and reduce the impact of the colored noise, which verifies the superiority of the proposed methods. The tracking accuracies of the proposed distributed algorithms are close to those of the centralized methods. This illustrates that the proposed distributed algorithms can obtain the global optimal estimation when a finite number of consensus iterations is performed. Finally, the differences between the tracking performances of the two filters are not remarkable, so the ACUIF-MD method is a preferable choice because it has lower dimension and is easier to implement. 

## Figures and Tables

**Table 1:** The initial condition of parameters in the simulation.

Terms	Values
Initial state error	$\Delta x_{0}=\left[1\mathrm{km};\;1\mathrm{km};\;1\mathrm{km};\;1m/s;\;1m/s;\;1m/s\right]$
Initial covariance matrix	$\sigma_{r,0}=1\mathrm{km},\;\sigma_{v,0}=1m/s$
Process noise matrix	$\sigma_{v,Q}=1e-2,\;\sigma_{a,Q}=1e-5$
Simulation duration	3000 s
Sampling step	1 s
$\sigma_{\rho }$	1 m
*L*	5
θ	0.25

**Table 2:** The initial positions and velocities of target and observation platforms.

	*x* (km)	*y* (km)	*z* (km)	*v_x_* (km/s)	*v_y_* (km/s)	*v_z_* (km/s)
Target	−251.66	2591.94	−6796.42	3.83	−5.87	−2.38
Observation platform 1	−117.92	2389.05	−6873.86	3.83	−5.96	−2.14
Observation platform 2	−368.43	2104.52	−6957.49	3.75	−6.05	−2.03
Observation platform 3	−496.83	2310.90	−6883.62	3.73	−5.97	−2.27
Observation platform 4	−434.62	2207.66	−6921.61	3.75	−6.01	−2.15
